# Correlation Between Pain Intensity in Different Locations and Intraoperative Stage of Endometriosis According to rASRM and #ENZIAN Classification

**DOI:** 10.3390/jcm15072725

**Published:** 2026-04-03

**Authors:** Krzysztof Przyśliwski, Maciej Pliszkiewicz, Joanna Jacko, Anna Bogaczyk, Bogumił Paweł Siekierski, Tomasz Kluz

**Affiliations:** 1 Faculty of Health Sciences and Psychology, University Center for Research and Development in Health Sciences, Collegium Medicum, University of Rzeszow, 35-310 Rzeszów, Poland; 2 Medicover Wilanów Hospital, Medicover Healthcare Services, 02-972 Warszawa, Poland; maciej.pliszkiewicz@medicover.pl (M.P.); joanna.jacko@medicover.pl (J.J.); bogumil.siekierski@medicover.pl (B.P.S.); 3 Department of Gynecology, Gynecology Oncology and Obstetrics, Faculty of Medicine, University of Rzeszow, 35-959 Rzeszów, Poland; annabogaczyk@interia.pl (A.B.); jtkluz@interia.pl (T.K.)

**Keywords:** endometriosis, pelvic pain, rASRM classification, #ENZIAN classification, pain assessment

## Abstract

**Background/Objectives:** Endometriosis is a chronic inflammatory disease with a heterogeneous clinical presentation, in which pain represents the predominant symptom. The association between pain severity and intraoperative disease stage remains unclear, particularly with regard to the rASRM and #ENZIAN classifications. This study aimed to evaluate the relationship between pain intensity at different anatomical sites and the stage of endometriosis according to the rASRM and #ENZIAN systems. **Methods**: A total of 138 patients with advanced endometriosis undergoing surgical treatment between May 2024 and August 2025 were included. Pain intensity was assessed using a 10-point Numerical Rating Scale (NRS) for pelvic pain, pain during defecation, pain during micturition, and pain during or after sexual intercourse. The stage of endometriosis was evaluated intraoperatively according to the rASRM and #ENZIAN classifications. Non-parametric statistical tests and Spearman’s rank correlation coefficient were applied. A *p*-value < 0.05 was considered statistically significant. **Results**: No significant correlation was found between overall pelvic pain intensity and disease stage according to the rASRM classification. However, significant differences in pain during micturition were observed depending on rASRM stage (*p* = 0.004). In the #ENZIAN-based analysis, significant associations were identified between selected anatomical areas and specific pain symptoms, particularly pain during micturition, defecation, and sexual intercourse. **Conclusions**: Pain severity in advanced endometriosis does not consistently correlate with overall disease stage according to rASRM. The anatomical localization of lesions defined by the #ENZIAN classification may better reflect the type and distribution of pain symptoms. These findings should be interpreted in the context of a selected cohort of surgically treated patients with advanced disease and may not be generalizable to patients with milder or non-surgically managed endometriosis.

## 1. Introduction

Endometriosis is a common chronic gynecological disease affecting approximately 10% of women of reproductive age. According to more recent data, endometriosis affects approximately 10–15% of women in this population [1,2] and up to 70% of women with persistent pelvic pain [3]. It is estimated that approximately 176–190 million women worldwide are affected by the disease [4,5], and nearly one-third of them experience infertility [6].

The age at diagnosis typically ranges from 20 to 45 years, with a reported median age of 28.8 years and mean age of 29.4 ± 7.7 years [7]. The disorder is characterized by the presence of endometrium-like tissue outside the uterine cavity, most commonly within the abdominal cavity, leading to a chronic inflammatory and proliferative response [8,9].

Long-term symptoms include cyclic and chronic pelvic pain, dyspareunia, dyschezia, dysuria, abnormal uterine bleeding, infertility, and fatigue [10]. Only 20–25% of patients with endometriosis remain asymptomatic, whereas up to 80% experience chronic pain and 30–50% face fertility-related difficulties [11,12].

The heterogeneity of symptoms, their variable severity, and the lack of specific diagnostic tools contribute to substantial delays in the diagnosis of endometriosis. The time from symptom onset to diagnosis may extend over several years and depends on factors such as population characteristics and diagnostic criteria. It can be broadly divided into the interval from symptom onset to first medical consultation and the time from initial consultation to confirmed diagnosis.

The multifaceted nature of the disease burden—including persistent pain during physiological activities such as defecation, micturition, and sexual intercourse, as well as menstrual irregularities and systemic symptoms—significantly affects patients’ psychological and social well-being [13,14].

For many patients, endometriosis is associated with reduced reproductive potential or infertility [15,16]. The chronic and cyclical nature of symptoms, combined with variable severity, impacts family life and relationships with children, partners, relatives, friends, and colleagues [17]. From an occupational perspective, the disease contributes to absenteeism, reduced productivity, and premature termination of employment, while also imposing a substantial burden on healthcare systems [18,19]. Patients frequently report that their symptoms are overlooked or trivialized—often dismissed as “just the way you are” [20] (p. 336)—which may lead to reduced trust in healthcare providers and social stigmatization [21]. Persistent symptoms and limited treatment effectiveness may further contribute to psychological distress, including anxiety and depressive symptoms [22].

A wide range of therapeutic approaches for endometriosis are available, including both conservative and surgical strategies. Conservative management encompasses nutritional interventions, psychological support, analgesic therapy, hormonal treatment, and physiotherapy, applied individually or in combination.

Surgical treatment, although often necessary, may be incomplete due to diagnostic challenges, inappropriate patient selection or insufficient expertise and competencies of surgical teams. In some cases, persistent endometriotic lesions are identified during repeat procedures. Moreover, even technically successful surgical intervention does not always result in improvement in physical, psychological, and social well-being, as reflected on quality-of-life assessments.

The aim of this study was to analyze the relationship between pain intensity reported by patients with endometriosis, assessed at different anatomical locations using an 11-point Numerical Rating Scale (NRS), and the intraoperative stage of the disease determined according to the rASRM and #ENZIAN classifications.

The specific objectives of this study were to assess the correlation between pain intensity (including pelvic pain, pain during defecation, micturition, and dyspareunia) and the stage of endometriosis, to evaluate the association between the number of pain locations and disease severity, and to analyze the relationship between involvement of specific compartments in the #ENZIAN classification and pain characteristics.

## 2. Materials and Methods

### 2.1. Study Population

The study included patients qualified for surgical removal of advanced deep endometriosis lesions confirmed by imaging and clinical diagnostics. Surgical procedures were performed between 1 May 2024, and 31 August 2025, at the Department of Gynecology, Medicover Hospital in Warsaw.

Perioperative diagnostics were primarily based on transvaginal ultrasound (TVUS), while magnetic resonance imaging (MRI) was performed in selected cases when additional assessment was required, due to inconclusive ultrasound findings.

A total of 138 consecutive patients with advanced endometriosis who were scheduled for surgical treatment at the study center during the defined study period were included in the study. The study population represents a selected cohort of patients with advanced endometriosis referred for a surgical treatment at a tertiary care center. The sample size was not determined by a priori power calculation; instead, the analyzed cohort represents a convenience sample reflecting real-world clinical practice. Given the exploratory nature of the study and the relatively limited sample size, no formal power analysis was performed. Therefore, the findings should be interpreted with caution and considered hypothesis-generating rather than confirmatory.

Prior to enrollment, all participants received written information about the purpose of the study and provided informed consent to participate. The questionnaire survey constituted part of a broader study evaluating pre- and postoperative quality of life in patients with endometriosis, which received approval from the Bioethics Committee of the Regional Medical Chamber in Warsaw (approval no. KB/1472/23).

Inclusion criteria comprised a diagnosis of deep endometriosis in patients qualified for surgical treatment, age over 18 years, and written informed consent to participate in the study. Exclusion criteria included age below 18 years, refusal to participate in the study, or disqualification from surgical treatment for any reason.

The following methods were applied: instruments for collecting sociodemographic data and medical history; tools for assessing pain intensity using the 10-point NRS; and classification systems for evaluating the stage of endometriosis—using the four-stage revised American Society for Reproductive Medicine classification (rASRM) and the #ENZIAN classification for deep endometriosis proposed by the Stiftung Endometriose-Forschung (SEF).

### 2.2. Data Collection

Sociodemographic data and detailed medical history were collected using a proprietary questionnaire developed specifically for the purposes of this study. Demographic variables included age, education level, employment status, and the predominant body position during work. Clinical variables included age, height, body weight, characteristics of the first menstrual period (date of onset, intensity, regularity, and associated pain), as well as the regularity, intensity, and pain associated with subsequent menstrual cycles.

Additional clinical data included the age at which endometriosis was diagnosed, the duration of symptoms prior to diagnosis, and the frequency, duration, and intensity of pain in the lower abdomen, during defecation, micturition, and during or after sexual intercourse. The questionnaire also included items assessing the impact of pain severity on daily functioning, the need for analgesic medication, the requirement for medical interventions due to pain, types of treatment received to date, and the number of surgical procedures related to endometriosis.

The Numerical Rating Scale (NRS) was also used to assess pain intensity associated with the main symptoms of the disease, including lower abdominal pain, pain during defecation, micturition, and dyspareunia. Owing to its high sensitivity, reliability, and reproducibility compared with other pain assessment tools, the NRS is widely used in clinical research [23]. The scale consists of 11 levels of pain intensity ranging from 0 to 10, where 0 indicates no pain and 10 represents the worst pain imaginable. The scale is characterized by a high level of reproducibility of results. For the purposes of analysis, pain intensity scores were categorized into the following groups: NRS score of “0”—no pain; “1–4”—mild pain; “5–7”—moderate pain; and “8–10”—severe pain.

The stage of endometriosis was determined intraoperatively by the surgical team of the Department of Gynecology using the rASRM and #ENZIAN classification systems [24,25]. Data used for this analysis were obtained from the operative reports of patients included in the study.

### 2.3. Statistical Analysis

Data obtained from the proprietary questionnaire, including the sociodemographic

Section and detailed interview data were entered into a specifically prepared Microsoft Excel spreadsheet and subsequently subjected to statistical analysis.

Quantitative variables were analyzed using descriptive statistics, including mean, standard deviation, median, quartiles, as well as minimum and maximum values, while qualitative variables were presented as absolute numbers and percentages.

Comparisons of qualitative variables between groups were performed using the chi-square test (with Yates’ correction for 2 × 2 contingency tables) or Fisher’s exact test when assumptions of the chi-square test were not met. Comparisons of quantitative variables between two groups were performed using the Mann–Whitney U test, while comparisons across three or more groups were performed using the Kruskal–Wallis test followed by Dunn’s post hoc test when appropriate. Correlations between quantitative variables were analyzed using Spearman’s rank correlation coefficient. The choice of non-parametric tests was based on the non-normal distribution of the analyzed variables.

A significance level of 0.05 was adopted for all analyses; therefore, *p*-values < 0.05 were considered indicative of statistical significance. Statistical analyses were performed using R software, version 4.5.1.

All patient personal data were anonymized. Correlations were analyzed between sociodemographic factors and clinical variables—including patient age, age at menarche, age at diagnosis, and the duration of symptoms prior to diagnosis of endometriosis to surgery—and the occurrence of pain in four anatomical locations: lower abdominal pain, pain during defecation, pain during micturition, and pain during or after sexual intercourse. These variables were also analyzed in relation to the intraoperative assessment of the disease stage according to the rASRM and #ENZIAN classification systems.

Given the large number of subgroup analyses performed across multiple #ENZIAN compartments and pain outcomes, no formal correction for multiple comparisons was applied. Therefore, the results should be interpreted with caution, and the findings should be considered exploratory rather than confirmatory.

## 3. Results

The following analyses should be interpreted as exploratory due to the number of subgroup comparisons performed.

### 3.1. Baseline Characteristics

A total of 138 patients with diagnosed advanced endometriosis, scheduled for elective surgical removal of endometriotic lesions at Medicover Hospital in Warsaw, were included in the study. Histological confirmation of endometriosis was obtained in all 138 patients. All participants met the inclusion criteria and provided informed consent to participate in the study.

### 3.2. Detailed Medical Interview

A total of 138 patients were included in the study, of whom 13 did not complete all items in the questionnaire section concerning aspects of sexual life. The corresponding missing data were considered in the baseline dataset used for statistical analyses.

The characteristics of patients with endometriosis are presented in Table 1. All subsequent analyses were performed within the same cohort, and therefore baseline characteristics were not repeated in the following tables to avoid redundancy.

### 3.3. Intraoperative Assessment of Endometriosis Severity

Disease severity was assessed using the rASRM and #ENZIAN classification systems (Table 2). For paired organs evaluated according to the #ENZIAN classification, if one side contained a numerical value and the other was marked as “m” or “x”, the numerical value was used for the analysis. For example, the notation “x_2” was recorded as “2”, whereas “0_m” was recorded as “0”. The entry “m_m” was reported as “m”.

In cases marked “T”, the patency test was omitted because this parameter was frequently not specified in the medical records (i.e., only the numeric value was recorded without indicating a positive or negative result).

### 3.4. Correlation Between Pain Intensity and the Number of Pain Locations and Disease Stage According to the rASRM and #ENZIAN Classifications

#### 3.4.1. rASRM Classification

**Figure 1:**
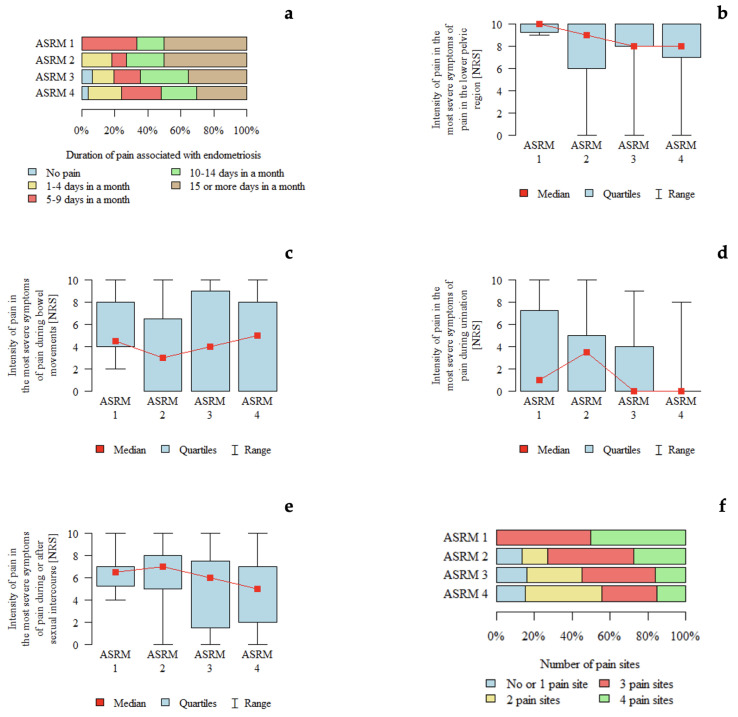
Pain characteristics according to the rASRM classification: (**a**) Duration of pain associated with endometriosis according to rASRM stage. (**b**) Intensity of pain in the lower pelvic region (NRS) according to rASRM stage. (**c**) Intensity of pain during bowel movements (NRS) according to rASRM stage. (**d**) Intensity of pain during urination (NRS) according to rASRM stage. (**e**) Intensity of pain during or after sexual intercourse (NRS) according to rASRM stage. (**f**) Number of pain locations according to rASRM stage.

*p*-values below 0.05 were considered indicative of statistically significant differences between groups. An association between pain intensity during micturition and rASRM stage was observed. Pain intensity appeared to be higher in patients with rASRM stage 2 compared with those with rASRM stage 3 and stage 4 (Table 3). A graphical representation of these findings is shown in Figure 1.

#### 3.4.2. ENZIAN P

The following analyses of #ENZIAN compartments are presented in a simplified and structured manner, with detailed results provided only for compartments showing statistically relevant associations, to improve clarity and avoid redundancy.

**Figure 2:**
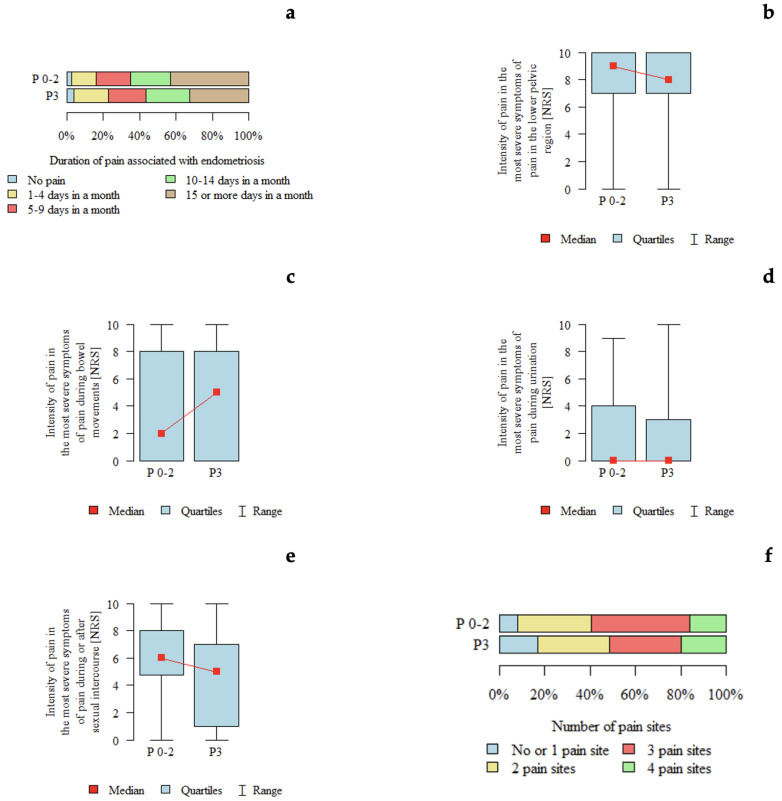
Pain characteristics according to the #ENZIAN P classification (peritoneal compartment): (**a**) Duration of pain associated with endometriosis according to #ENZIAN P stage. (**b**) Intensity of pain in the lower pelvic region (NRS) according to #ENZIAN P stage. (**c**) Intensity of pain during bowel movements (NRS) according to #ENZIAN P stage. (**d**) Intensity of pain during urination (NRS) according to #ENZIAN P stage. (**e**) Intensity of pain during or after sexual intercourse (NRS) according to #ENZIAN P stage. (**f**) Number of pain locations according to #ENZIAN P stage.

*p*-values below 0.05 indicate statistically significant differences between groups. An association between ENZIAN P classification and pain intensity during or after sexual intercourse was observed. Pain intensity appeared to be higher in patients with P0–P2 compared with those with P3. The detailed distribution of pain intensity according to #ENZIAN P classification is presented in Table 4 and Figure 2.

#### 3.4.3. ENZIAN O

**Figure 3:**
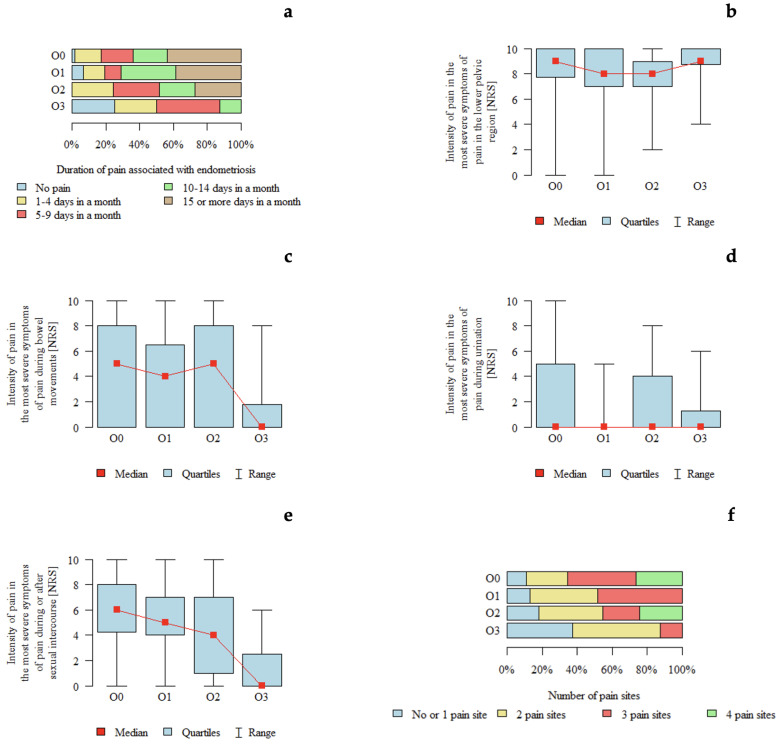
Pain characteristics according to the #ENZIAN O classification (ovarian compartment): (**a**) Duration of pain associated with endometriosis according to #ENZIAN O stage. (**b**) Intensity of pain in the lower pelvic region (NRS) according to #ENZIAN O stage. (**c**) Intensity of pain during bowel movements (NRS) according to #ENZIAN O stage. (**d**) Intensity of pain during urination (NRS) according to #ENZIAN O stage. (**e**) Intensity of pain during or after sexual intercourse (NRS) according to #ENZIAN O stage. (**f**) Number of pain locations according to #ENZIAN O stage.

*p*-values below 0.05 indicate statistically significant differences between groups. The results are presented in Table 5 and Figure 3. An association between #ENZIAN O classification and pain characteristics was observed. Pain symptoms appeared to be more frequent in patients with O0 and less frequent in those with O3. Pain intensity during micturition appeared to be higher in patients with O0 compared with those with O1. Similarly, pain during or after sexual intercourse appeared to be higher in patients with O0–O2 compared with those with O3. The number of pain locations also appeared to be higher in patients with O0 and lower in those with O3.

#### 3.4.4. ENZIAN T

**Figure 4:**
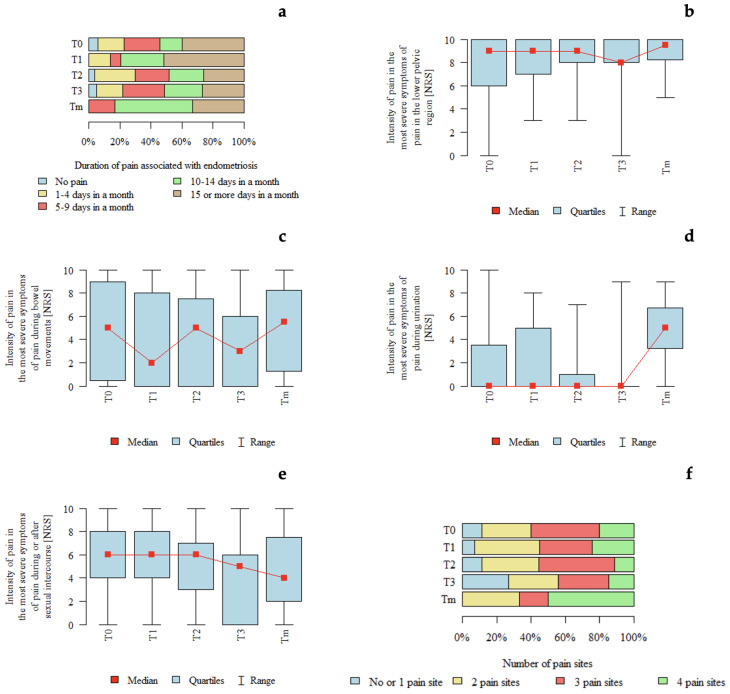
Pain characteristics according to the #ENZIAN T classification (tubal compartment): (**a**) Duration of pain associated with endometriosis according to #ENZIAN T stage. (**b**) Intensity of pain in the lower pelvic region (NRS) according to #ENZIAN T stage. (**c**) Intensity of pain during bowel movements (NRS) according to #ENZIAN T stage. (**d**) Intensity of pain during urination (NRS) according to #ENZIAN T stage. (**e**) Intensity of pain during or after sexual intercourse (NRS) according to #ENZIAN T stage. (**f**) Number of pain locations according to #ENZIAN T stage.

*p*-values below 0.05 indicate statistically significant differences between groups. An association between #ENZIAN T classification and pain intensity during micturition was observed. Pain intensity appeared to be higher in patients with Tm compared with those with T1, T0, T2, and T3, and higher in patients with T1 compared with those with T3 (Table 6). The graphical distribution of pain characteristics according to the #ENZIAN T classification is presented in Figure 4.

#### 3.4.5. ENZIAN A

No statistically significant associations were observed for the ENZIAN A compartment (all *p*-values > 0.05).

#### 3.4.6. ENZIAN B

**Figure 5:**
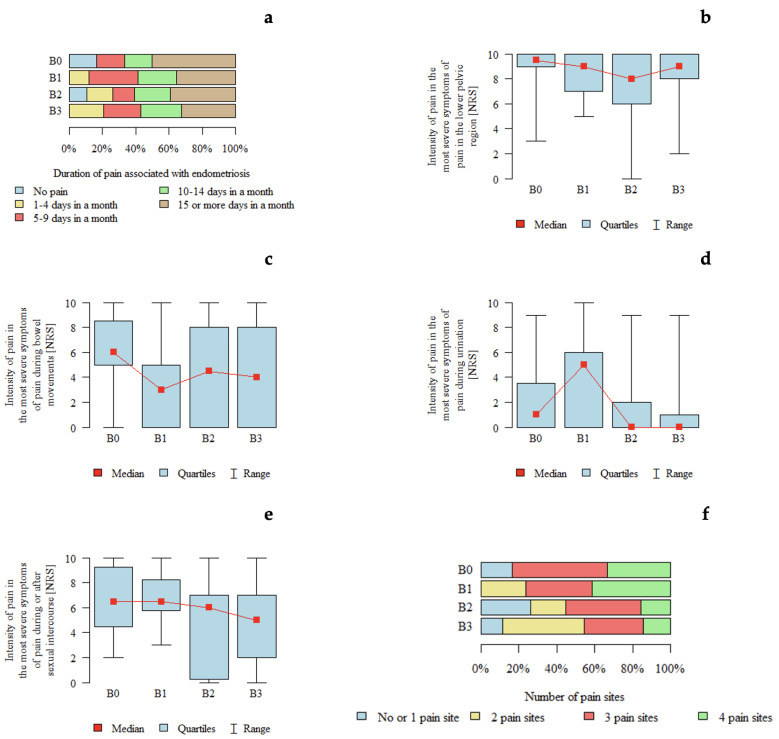
Pain characteristics according to the #ENZIAN B classification (uterosacral ligaments and pelvic sidewall): (**a**) Duration of pain associated with endometriosis according to #ENZIAN B stage. (**b**) Intensity of pain in the lower pelvic region (NRS) according to #ENZIAN B stage. (**c**) Intensity of pain during bowel movements (NRS) according to #ENZIAN B stage. (**d**) Intensity of pain during urination (NRS) according to #ENZIAN B stage. (**e**) Intensity of pain during or after sexual intercourse (NRS) according to #ENZIAN B stage. (**f**) Number of pain locations according to #ENZIAN B stage.

*p*-values below 0.05 indicate statistically significant differences between groups. The results are presented in Table 7 and Figure 5. An association between #ENZIAN B classification and pain characteristics was observed. Pain intensity during micturition appeared to be higher in patients with B1 compared with those with B2 and B3. The number of pain locations also appeared to be higher in patients with B1 and lower in those with B2.

#### 3.4.7. ENZIAN C

**Figure 6:**
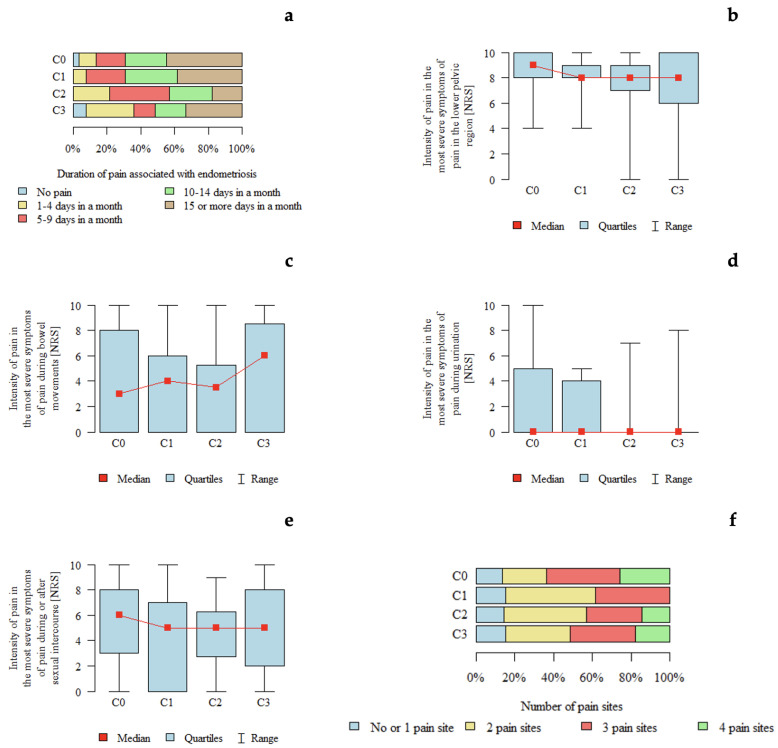
Pain characteristics according to the #ENZIAN C classification (rectal compartment): (**a**) Duration of pain associated with endometriosis according to #ENZIAN C stage. (**b**) Intensity of pain in the lower pelvic region (NRS) according to #ENZIAN C stage. (**c**) Intensity of pain during bowel movements (NRS) according to #ENZIAN C stage. (**d**) Intensity of pain during urination (NRS) according to #ENZIAN C stage. (**e**) Intensity of pain during or after sexual intercourse (NRS) according to #ENZIAN C stage. (**f**) Number of pain locations according to #ENZIAN C stage.

*p*-values below 0.05 indicate statistically significant differences between groups. An association between #ENZIAN C classification and pain intensity during micturition was observed. Pain intensity appeared to be higher in patients with C0 compared with those with C3 and C2 (Table 8). The graphical representation of pain characteristics according to the #ENZIAN C is shown in Figure 6.

#### 3.4.8. FA Adenomiosis

No statistically significant associations were observed for this ENZIAN compartment (all *p*-values > 0.05).

#### 3.4.9. FB Bladder

**Figure 7:**
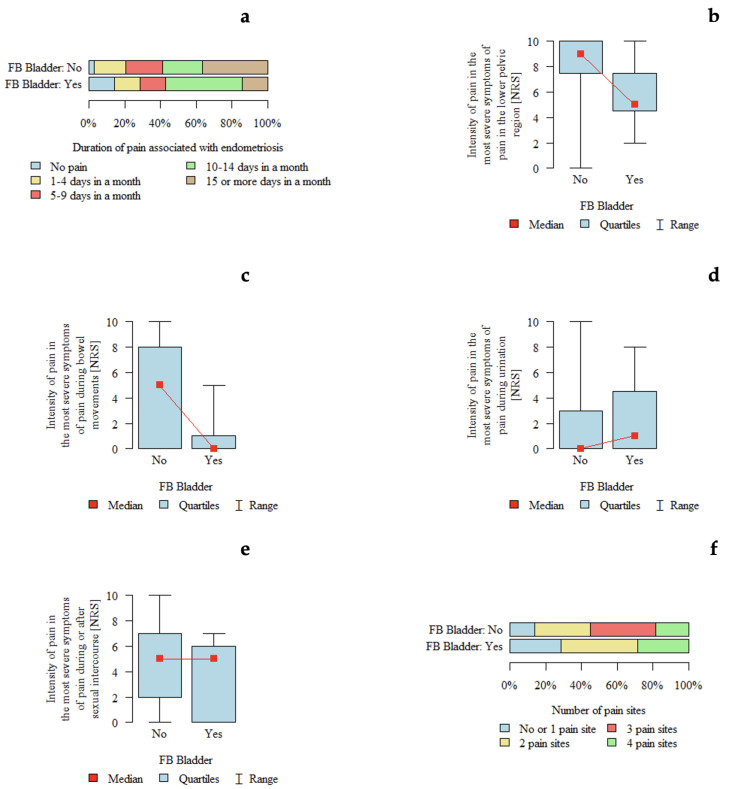
Pain characteristics according to bladder involvement (FB in the #ENZIAN classification): (**a**) Duration of pain associated with endometriosis according to bladder involvement. (**b**) Intensity of pain in the lower pelvic region (NRS). (**c**) Intensity of pain during bowel movements (NRS). (**d**) Intensity of pain during urination (NRS). (**e**) Intensity of pain during or after sexual intercourse (NRS). (**f**) Number of pain locations.

*p*-values below 0.05 indicate statistically significant differences between groups. The results are presented in Table 9 and Figure 7. An association between #ENZIAN FB classification and pain characteristics was observed. Pain intensity in the lower pelvic region appeared to be higher in patients without FB compared with those with FB. Similarly, pain intensity during defecation appeared to be higher in patients without FB compared with those with FB.

#### 3.4.10. FI Intestine

**Figure 8:**
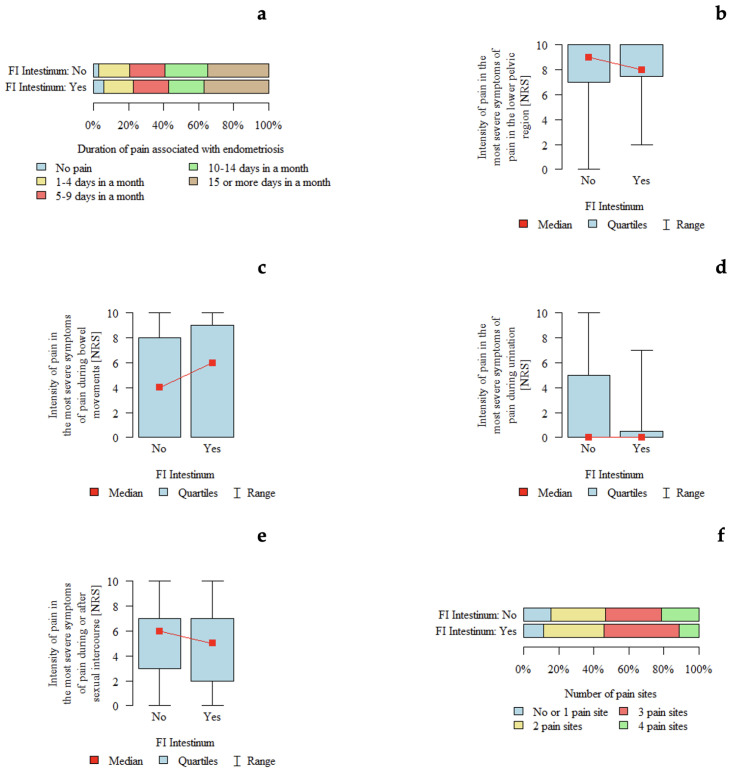
Pain characteristics according to intestinal involvement (FI in the #ENZIAN classification): (**a**) Duration of pain associated with endometriosis according to intestinal involvement. (**b**) Intensity of pain in the lower pelvic region (NRS). (**c**) Intensity of pain during bowel movements (NRS). (**d**) Intensity of pain during urination (NRS). (**e**) Intensity of pain during or after sexual intercourse (NRS). (**f**) Number of pain locations.

*p*-values below 0.05 indicate statistically significant differences between groups. An association between intestinal involvement (#ENZIAN FI) and pain intensity during defecation was observed. Pain intensity appeared to be higher in patients with FI compared with those without FI (Table 10). The graphical presentation of pain characteristics according to intestinal involvement is shown in Figure 8.

#### 3.4.11. FD Diaphragm

No statistically significant associations were observed for this ENZIAN compartment (all *p*-values > 0.05).

## 4. Discussion

Endometriosis affects women across a wide age range, most commonly between 20 and 40 years of age; however, it may also occur in adolescents and postmenopausal women [26,27]. The time from the onset of symptoms to diagnosis varies considerably and has been reported to range from approximately 0.3 to 12 years [13].

The findings obtained should be interpreted with caution and considered exploratory rather than definitive.

In our study, the mean age of patients at the time of endometriosis diagnosis was 32.19 years. More than 70% of patients reported experiencing symptoms of endometriosis for over 7 years, with 59.42% indicating a duration of symptoms exceeding 10 years. In contrast, fewer than 14% of patients reported symptom duration of less than 3 years.

Endometriosis is an estrogen-dependent disease that affects women across a wide age range. Due to its chronic nature, variable symptom severity, and wide spectrum of clinical manifestations, endometriosis significantly affects patients’ quality of life. Pain symptoms, often associated with the multi-organ localization of endometriotic lesions, may substantially impair patients’ functioning in their private, professional, and social lives.

Our findings suggest that pain symptoms in endometriosis may be more closely related to lesion localization than to overall disease stage. While the rASRM classification provides a general assessment of disease severity, it may not adequately reflect symptom-specific burden. In contrast, the #ENZIAN classification, by describing compartment-specific involvement, may offer a more clinically relevant framework for understanding pain patterns.

In our study, a positive correlation was observed between pain intensity during or after sexual intercourse and disease stage according to the #ENZIAN classification in the P (peritoneal) compartment, with higher pain intensity reported among patients with stages P0-P2 compared with P3.

In the O (ovarian) compartment, a positive association was observed between pain intensity and lower stages of ovarian involvement. Higher pain intensity was reported among patients with absent or less extensive ovarian lesions compared with those with more advanced ovarian involvement. Specifically, higher pain intensity was observed in patients with O0 compared with O1 for pelvic and pain during micturition, and in patients with O0–O2 compared with O3 for pain during or after sexual intercourse. Additionally, the number of pain locations was highest among patients with O0 compared with patients with O3.

In the T (tubal) compartment, a positive association was observed between pain intensity during micturition and the presence of minimal or unspecified tubal involvement. Higher pain intensity during micturition was reported among patients classified as Tm compared with patients with lesions classified as T1–T3.

No significant associations were found between the number of pain locations, duration of pain symptoms related to endometriosis, or pain intensity at the reported location, and the presence of endometriotic lesions in the rectovaginal, rectocervical, and vaginal compartments according to #ENZIAN A classification (A0–A3).

The presence of endometriotic lesions in the uterosacral ligaments and pelvic sidewall, corresponding to the #ENZIAN B compartment, showed a positive association between pain intensity during micturition and less advanced disease stages. Specifically, pain intensity during micturition was significantly higher in patients with B1 compared with those with B2-B3. Similarly, the number of pain locations reported by patients was higher in cases where the intraoperative disease stage was classified as B1 compared with B2 and B3.

Interestingly, some patients with less extensive disease appeared to report higher pain intensity. This may be related to lesion localization rather than overall disease extent. Lesions affecting highly innervated structures or areas exposed to mechanical stress may generate more pronounced symptoms. Additionally, mechanisms such as peripheral nerve involvement and central sensitization may contribute to pain perception independently of anatomical disease severity.

However, some of the observed associations, particularly those in which less extensive disease or the absence of involvement in specific compartments was associated with higher pain intensity, should be interpreted with caution. These findings may reflect small subgroup sizes, overlapping lesion distribution, or heterogeneity of symptom presentation rather than true pathophysiological relationships. Additionally, methodological factors, including the exploratory nature of the analysis and the lack of adjustment for multiple comparisons, may have contributed to these observations. Therefore, direct anatomical interpretations of such results should be avoided.

For endometriotic lesions located in the rectal compartment (#ENZIAN C), a statistically significant association was observed with pain intensity during micturition. Pain during micturition was reported significantly more frequently among patients without rectal lesions compared with those with rectal involvement (C2–C3).

Endometriotic lesions located in the bladder (#ENZIAN FB) were associated with differences in the perception of pain in the lower pelvic region and during defecation. Higher pain intensity in these domains was observed among patients without bladder involvement compared with those with lesions located in the bladder.

This observation may suggest that the presence of bladder lesions shifts the perception of pain toward symptoms more specifically related to the anterior compartment, such as bladder-associated discomfort. Consequently, the perception of more generalized pelvic pain or pain associated with defecation may become relatively less pronounced, while symptoms originating from the bladder itself become more prominent.

From a clinical perspective, these findings may have implications for surgical planning. A compartment-based classification such as #ENZIAN may help identify lesions more likely to be associated with specific pain symptoms, potentially supporting more targeted surgical approaches. These findings may support a more symptom-oriented surgical approach, in which lesion localization rather than overall disease stage is considered during preoperative planning.

In cases of endometriotic lesions involving the intestinal compartment (#ENZIAN FI), a positive association was observed between pain intensity during defecation and the presence of intestinal involvement. This finding may be explained by the anatomical characteristics of this compartment. In the #ENZIAN classification, FI primarily refers to lesions located in the large intestine above the rectum, which represents a relatively common site of deep endometriosis.

In contrast, involvement of the small intestine, particularly the terminal ileum, is considerably less frequent and was observed only in a few cases in the analyzed material, as the FI compartment.

No statistically significant differences were observed in the number of pain locations, pain intensity, or duration of pain in cases of endometriotic lesions located in the diaphragmatic compartment (#ENZIAN FD).

It should also be noted that some statistically significant findings, such as the association between pain during urination in the rASRM analysis and dyspareunia in the #ENZIAN P compartment, may not remain significant after correction for multiple comparisons.

This study has several limitations. The retrospective design and inclusion of highly selected cohort of surgically treated patients with advanced endometriosis limit the generalizability and external validity of the findings. The large number of subgroup analyses without formal correction for multiple comparisons increases the risk of Type I error; therefore, the results should be interpreted as exploratory and hypothesis-generating rather than confirmatory. Additionally, small subgroup sizes may have affected the robustness of the observed associations.

The use of Numeric Rating Scale (NRS) represents a limitation, as it provides a unidimensional assessment of pain and does not capture its qualitative, emotional, or functional aspects. Furthermore, the lack of detailed analysis of concomitant diseases, which may influence pain perception and clinical presentation, constitutes an additional limitation. Finally, the interpretation of results was primarily based on *p*-values without reporting effect sizes or confidence intervals, which may limit the assessment of the magnitude and clinical relevance of the observed associations.

## 5. Conclusions

Despite increasing diagnostic and therapeutic possibilities, endometriosis remains a chronic condition that significantly impairs the quality of life of affected women for many years. The heterogenous course of the disease and the variability and multiplicity of symptoms mean that the clinical presentation often does not clearly reflect the intraoperative stage of disease severity.

In the present analysis, no clear correlation was observed between overall pelvic pain intensity and the stage of endometriosis according to the rASRM classification. However, the results suggest that the relationship between pain symptoms and intraoperative findings becomes more apparent when the specific anatomical localization of lesions is analyzed according to the #ENZIAN classification. This indicates that the location and character of endometriotic lesions may have greater clinical relevance for the type and intensity of pain than the global stage of disease severity.

The findings highlight the importance of individualized diagnostic and therapeutic approaches, as well as the need to consider the subjective experience of pain as a key element of clinical assessment.

At the same time, the multifaceted impact of endometriosis on the physical, psychological, and social functioning of patients underscores the need to strengthen educational initiatives, increase health awareness, and provide holistic care for women with suspected or confirmed endometriosis.

## Figures and Tables

**Table 1:** Patients’ characteristics.

Parameter		Total (N = 138)
Age [years]	Mean (SD)	37.22 (6.58)
	Median (quartiles)	37 (33–42)
	Range	22–54
	n	138
BMI	Underweight	10 (7.25%)
	Normal weight	91 (65.94%)
	Overweight	27 (19.57%)
	Obesity	10 (7.25%)
Education	Primary	1 (0.72%)
	Vocational	7 (5.07%)
	Secondary	14 (10.14%)
	Higher	116 (84.06%)
Professional activity	Student	1 (0.72%)
	Employed	120 (86.96%)
	Unemployed	16 (11.59%)
	Pensioner	1 (0.72%)
Age at first menstruation [years]	Mean (SD)	12.88 (1.49)
	Median (quartiles)	13 (12–14)
	Range	9–17
	n	138
First menstruation	9–11 years	19 (13.77%)
	12–14 years	99 (71.74%)
	15–17 years	20 (14.49%)
Regularity of first menstruations	Initially regular, irregular over time	22 (15.94%)
	Initially irregular, regular over time	40 (28.99%)
	Always regular	58 (42.03%)
	Always irregular	18 (13.04%)
Age at diagnosis of endometriosis [years]	Mean (SD)	32.19 (6.91)
	Median (quartiles)	31 (27–37)
	Range	18–52
	n	138
Duration of symptoms currently associated with endometriosis	Less than 12 months	1 (0.72%)
	1–3 years	18 (13.04%)
	4–6 years	20 (14.49%)
	7–9 years	17 (12.32%)
	10 years or more	82 (59.42%)
Time from reporting the first symptoms to a doctor to diagnosis of endometriosis	Less than 12 months	19 (13.77%)
	1–3 years	27 (19.57%)
	4–6 years	20 (14.49%)
	7–9 years	23 (16.67%)
	10 years or more	49 (35.51%)
Circumstances in which pain is most commonly experienced *	No pain	3 (2.17%)
	Before menstruation	83 (60.14%)
	During menstruation	107 (77.54%)
	In the middle of the cycle	77 (55.80%)
	At various times, regardless of the phase of the cycle	70 (50.72%)
	In connection with physiological activities	80 (57.97%)
	During or after sexual intercourse	100 (72.46%)
Duration of pain associated with endometriosis	No pain	5 (3.62%)
	1–4 days in a month	24 (17.39%)
	5–9 days in a month	28 (20.29%)
	10–14 days in a month	32 (23.19%)
	15 or more days in a month	49 (35.51%)
Pain in the lower pelvic area	No	3 (2.17%)
	Yes	135 (97.83%)
Intensity of pain in the most severe symptoms of pain in the lower pelvic region [NRS]	Mean (SD)	8.09 (2.18)
	Median (quartiles)	9 (7–10)
	Range	0–10
	n	138
Intensity of pain in the most severe symptoms of pain in the lower pelvic region	No pain (0)	3 (2.17%)
	Mild pain (1–4)	6 (4.35%)
	Medium pain (5–7)	29 (21.01%)
	Severe pain (8–10)	100 (72.46%)
Intensity of pain in the most severe symptoms of pain during bowel movements [NRS]	Mean (SD)	4.17 (3.85)
	Median (quartiles)	4 (0–8)
	Range	0–10
	n	138
Intensity of pain in the most severe symptoms of pain during bowel movements	No pain (0)	52 (37.68%)
	Mild pain (1–4)	19 (13.77%)
	Medium pain (5–7)	26 (18.84%)
	Severe pain (8–10)	41 (29.71%)
Pain during urination	No	99 (71.74%)
	Yes	39 (28.26%)
Intensity of pain in the most severe symptoms of pain during urination [NRS]	Mean (SD)	1.76 (2.89)
	Median (quartiles)	0 (0–3.75)
	Range	0–10
	n	138
Intensity of pain in the most severe symptoms of pain during urination	No pain (0)	93 (67.39%)
	Mild pain (1–4)	14 (10.14%)
	Medium pain (5–7)	21 (15.22%)
	Severe pain (8–10)	10 (7.25%)
Pain during or after sexual intercourse	No	34 (24.64%)
	Yes	102 (73.91%)
	Unknown	2 (1.45%)
Intensity of pain in the most severe symptoms of pain during or after sexual intercourse [NRS]	Mean (SD)	4.9 (3.19)
	Median (quartiles)	5 (2–7)
	Range	0–10
	n	136
Intensity of pain in the most severe symptoms of pain during or after sexual intercourse	No pain (0)	26 (18.84%)
	Mild pain (1–4)	25 (18.12%)
	Medium pain (5–7)	54 (39.13%)
	Severe pain (8–10)	31 (22.46%)
	Unknown	2 (1.45%)
Other locations of pain *	Breasts	45 (32.61%)
	Legs	59 (42.75%)
	Lower back, spine area	114 (82.61%)
	Shoulders, arms	33 (23.91%)
	Chest	25 (18.12%)

* Multiple responses allowed; therefore, percentages do not sum to 100%.

**Table 2:** Intraoperative Staging of Endometriosis Severity According to the rASRM and #ENZIAN Classifications.

Parameter		Total (N = 138)
rASRM	rASRM 1	6 (4.35%)
	rASRM 2	22 (15.94%)
	rASRM 3	31 (22.46%)
	ASRM 4	79 (57.25%)
#Enzian P	P0	1 (0.72%)
	P1	3 (2.17%)
	P2	33 (23.91%)
	P3	101 (73.19%)
#Enzian O	O0	64 (46.38%)
	O1	31 (22.46%)
	O2	33 (23.91%)
	O3	8 (5.80%)
	Om	2 (1.45%)
#Enzian T	T0	35 (25.36%)
	T1	29 (21.01%)
	T2	27 (19.57%)
	T3	41 (29.71%)
	Tm	6 (4.35%)
#Enzian A	A0	14 (10.14%)
	A1	38 (27.54%)
	A2	31 (22.46%)
	A3	55 (39.86%)
#Enzian B	B0	6 (4.35%)
	B1	17 (12.32%)
	B2	38 (27.54%)
	B3	77 (55.80%)
#Enzian C	C0	58 (42.03%)
	C1	13 (9.42%)
	C2	28 (20.29%)
	C3	39 (28.26%)
#Enzian F *	FA Adenomyosis	107 (77.54%)
	FB Bladder	7 (5.07%)
	FI Intestinum	35 (25.36%)
	FU Ureter	3 (2.17%)
	FC Caecum	1 (0.72%)
	FD Diaphragm	6 (4.35%)

* Multiple responses allowed; therefore, percentages do not sum to 100%.

**Table 3:** Distribution of endometriosis stages according to the rASRM classification.

Parameter		ASRM 1 (N = 6)	ASRM 2 (N = 22)	ASRM 3 (N = 31)	ASRM 4 (N = 79)	*p*
Duration of pain associated with endometriosis	No pain	0 (0.00%)	0 (0.00%)	2 (6.45%)	3 (3.80%)	*p* = 0.697
	1–4 days in a month	0 (0.00%)	4 (18.18%)	4 (12.90%)	16 (20.25%)	
	5–9 days in a month	2 (33.33%)	2 (9.09%)	5 (16.13%)	19 (24.05%)	
	10–14 days in a month	1 (16.67%)	5 (22.73%)	9 (29.03%)	17 (21.52%)	
	15 or more days in a month	3 (50.00%)	11 (50.00%)	11 (35.48%)	24 (30.38%)	
Intensity of pain in the most severe symptoms of pain in the lower pelvic region [NRS]	Mean (SD)	9.67 (0.52)	7.73 (2.68)	8.32 (2.01)	7.99 (2.14)	*p* = 0.153
	Median (quartiles)	10 (9.25–10)	9 (6–10)	8 (8–10)	8 (7–10)	
	Range	9–10	0–10	0–10	0–10	
	n	6	22	31	79	
Intensity of pain in the most severe symptoms of pain during bowel movements [NRS]	Mean (SD)	5.67 (3.14)	3.45 (3.71)	4.45 (4.37)	4.14 (3.75)	*p* = 0.548
	Median (quartiles)	4.5 (4–8)	3 (0–6.5)	4 (0–9)	5 (0–8)	
	Range	2–10	0–10	0–10	0–10	
	n	6	22	31	79	
Intensity of pain in the most severe symptoms of pain during urination [NRS]	Mean (SD)	3.5 (4.72)	3.5 (3.41)	1.68 (2.81)	1.18 (2.38)	*p* = 0.004 * ASRM 2 > ASRM 3, ASRM 4
	Median (quartiles)	1 (0–7.25)	3.5 (0–5)	0 (0–4)	0 (0–0)	
	Range	0–10	0–10	0–9	0–8	
	n	6	22	31	79	
Intensity of pain in the most severe symptoms of pain during or after sexual intercourse [NRS]	Mean (SD)	6.5 (2.07)	6.1 (2.91)	4.87 (3.44)	4.47 (3.15)	*p* = 0.102
	Median (quartiles)	6.5 (5.25–7)	7 (5–8)	6 (1.5–7.5)	5 (2–7)	
	Range	4–10	0–10	0–10	0–10	
	n	6	21	31	78	
Number of pain sites	No or 1 pain site	0 (0.00%)	3 (13.64%)	5 (16.13%)	12 (15.19%)	*p* = 0.134
	2 pain sites	0 (0.00%)	3 (13.64%)	9 (29.03%)	32 (40.51%)	
	3 pain sites	3 (50%)	10 (45.45%)	12 (38.71%)	23 (29.11%)	
	4 pain sites	3 (50%)	6 (27.27%)	5 (16.13%)	12 (15.19%)	

*p*—Qualitative variables: chi-squared or Fisher’s exact test. Quantitative variables: Kruskal–Wallis test + post hoc analysis (Dunn test). * Statistically significant (*p* < 0.05).

**Table 4:** Pain characteristics according to the #ENZIAN P classification.

Parameter		P 0-2 (N = 37)	P3 (N = 101)	*p*
Duration of pain associated with endometriosis	No pain	1 (2.70%)	4 (3.96%)	*p* = 0.87
	1–4 days in a month	5 (13.51%)	19 (18.81%)	
	5–9 days in a month	7 (18.92%)	21 (20.79%)	
	10–14 days in a month	8 (21.62%)	24 (23.76%)	
	15 or more days in a month	16 (43.24%)	33 (32.67%)	
Intensity of pain in the most severe symptoms of pain in the lower pelvic region [NRS]	Mean (SD)	8 (2.6)	8.13 (2.01)	*p* = 0.693
	Median (quartiles)	9 (7–10)	8 (7–10)	
	Range	0–10	0–10	
	n	37	101	
Intensity of pain in the most severe symptoms of pain during bowel movements [NRS]	Mean (SD)	3.7 (4.05)	4.34 (3.78)	*p* = 0.401
	Median (quartiles)	2 (0–8)	5 (0–8)	
	Range	0–10	0–10	
	n	37	101	
Intensity of pain in the most severe symptoms of pain during urination [NRS]	Mean (SD)	2.11 (3.03)	1.63 (2.84)	*p* = 0.3
	Median (quartiles)	0 (0–4)	0 (0–3)	
	Range	0–9	0–10	
	n	37	101	
Intensity of pain in the most severe symptoms of pain during or after sexual intercourse [NRS]	Mean (SD)	5.92 (2.92)	4.54 (3.21)	*p* = 0.031 *
	Median (quartiles)	6 (4.75–8)	5 (1–7)	
	Range	0–10	0–10	
	n	36	100	
Number of pain sites	No or 1 pain site	3 (8,.11%)	17 (16.83%)	*p* = 0.448
	2 pain sites	12 (32.43%)	32 (31.68%)	
	3 pain sites	16 (43.24%)	32 (31.68%)	
	4 pain sites	6 (16.22%)	20 (19.80%)	

*p*—Qualitative variables: chi-squared or Fisher’s exact test. Quantitative variables: Mann–Whitney test. * Statistically significant (*p* < 0.05).

**Table 5:** Pain characteristics according to the #ENZIAN O classification.

Parameter		O0 (N = 64)	O1 (N = 31)	O2 (N = 33)	O3 (N = 8)	*p*
Duration of pain associated with endometriosis	No pain	1 (1.56%)	2 (6.45%)	0 (0.00%)	2 (25.00%)	*p* = 0.018 *
	1–4 days in a month	10 (15.62%)	4 (12.90%)	8 (24.24%)	2 (25.00%)	
	5–9 days in a month	12 (18.75%)	3 (9.68%)	9 (27.27%)	3 (37.50%)	
	10–14 days in a month	13 (20.31%)	10 (32.26%)	7 (21.21%)	1 (12.50%)	
	15 or more days in a month	28 (43.75%)	12 (38.71%)	9 (27.27%)	0 (0.00%)	
Intensity of pain in the most severe symptoms of pain in the lower pelvic region [NRS]	Mean (SD)	8.27 (2.35)	7.81 (2.39)	7.85 (1.7)	8.62 (2)	*p* = 0.132
	Median (quartiles)	9 (7.75–10)	8 (7–10)	8 (7–9)	9 (8.75–10)	
	Range	0–10	0–10	2–10	4–10	
	n	64	31	33	8	
Intensity of pain in the most severe symptoms of pain during bowel movements [NRS]	Mean (SD)	4.67 (3.93)	3.77 (3.8)	3.97 (3.78)	1.62 (2.92)	*p* = 0.19
	Median (quartiles)	5 (0–8)	4 (0–6.5)	5 (0–8)	0 (0–1.75)	
	Range	0–10	0–10	0–10	0–8	
	n	64	31	33	8	
Intensity of pain in the most severe symptoms of pain during urination [NRS]	Mean (SD)	2.45 (3.37)	0.45 (1.31)	1.67 (2.65)	1.38 (2.56)	*p* = 0.028 * O0 > O1
	Median (quartiles)	0 (0–5)	0 (0–0)	0 (0–4)	0 (0–1.25)	
	Range	0–10	0–5	0–8	0–6	
	n	64	31	33	8	
Intensity of pain in the most severe symptoms of pain during or after sexual intercourse [NRS]	Mean (SD)	5.53 (3.21)	5.26 (2.74)	4.27 (3.23)	1.5 (2.33)	*p* = 0.007 * O0, O1, O2 > O3
	Median (quartiles)	6 (4.25–8)	5 (4–7)	4 (1–7)	0 (0–2.5)	
	Range	0–10	0–10	0–10	0–6	
	n	62	31	33	8	
Number of pain sites	No or 1 pain site	7 (10.94%)	31 (100.00%)	6 (18.18%)	3 (37.50%)	*p* = 0.01*
	2 pain sites	15 (23.44%)	12 (38.71%)	12 (36.36%)	4 (50.00%)	
	3 pain sites	25 (39.06%)	15 (48.39%)	7 (21.21%)	1 (12.50%)	
	4 pain sites	17 (26.56%)	0 (0.00%)	8 (24.24%)	0 (0.00%)	

*p*—Qualitative variables: chi-squared or Fisher’s exact test. Quantitative variables: Kruskal–Wallis test + post hoc analysis (Dunn test). * Statistically significant (*p* < 0.05).

**Table 6:** Pain characteristics according to the #ENZIAN T classification.

Parameter		T0 (N = 35)	T1 (N = 29)	T2 (N = 27)	T3 (N = 41)	Tm (N = 6)	*p*
Duration of pain associated with endometriosis	No pain	2 (5.71%)	0 (0.00%)	1 (3.70%)	2 (4.88%)	0 (0.00%)	*p* = 0.514
	1–4 days in a month	6 (17.14%)	4 (13.79%)	7 (25.93%)	7 (17.07%)	0 (0.00%)	
	5–9 days in a month	8 (22.86%)	2 (6.90%)	6 (22.22%)	11 (26.83%)	1 (16.67%)	
	10–14 days in a month	5 (14.29%)	8 (27.59%)	6 (22.22%)	10 (24.39%)	3 (50.00%)	
	15 or more days in a month	14 (40.00%)	15 (51.72%)	7 (25.93%)	11 (26.83%)	2 (33.33%)	
Intensity of pain in the most severe symptoms of pain in the lower pelvic region [NRS]	Mean (SD)	7.6 (2.87)	8.17 (2.07)	8.37 (1.71)	8.2 (1.87)	8.67 (1.97)	*p* = 0.9
	Median (quartiles)	9 (6–10)	9 (7–10)	9 (8–10)	8 (8–10)	9.5 (8.25–10)	
	Range	0–10	3–10	3–10	0–10	5–10	
	n	35	29	27	41	6	
Intensity of pain in the most severe symptoms of pain during bowel movements [NRS]	Mean (SD)	5.17 (3.99)	3.55 (3.87)	4.37 (3.62)	3.49 (3.78)	5 (4.29)	*p* = 0.285
	Median (quartiles)	5 (0.5–9)	2 (0–8)	5 (0–7.5)	3 (0–6)	5.5 (1.25–8.25)	
	Range	0–10	0–10	0–10	0–10	0–10	
	n	35	29	27	41	6	
Intensity of pain in the most severe symptoms of pain during urination [NRS]	Mean (SD)	2.11 (3.38)	2.21 (2.93)	1.3 (2.33)	1 (2.38)	4.83 (3.19)	*p* = 0.012 * Tm > T1, T0, T2, T3 T1 > T3
	Median (quartiles)	0 (0–3.5)	0 (0–5)	0 (0–1)	0 (0–0)	5 (3.25–6.75)	
	Range	0–10	0–8	0–7	0–9	0–9	
	n	35	29	27	41	6	
Intensity of pain in the most severe symptoms of pain during or after sexual intercourse [NRS]	Mean (SD)	5.39 (3.12)	5.55 (3.11)	5 (3.03)	4.02 (3.24)	4.67 (3.93)	*p* = 0.265
	Median (quartiles)	6 (4–8)	6 (4–8)	6 (3–7)	5 (0–6)	4 (2–7.5)	
	Range	0–10	0–10	0–10	0–10	0–10	
	n	33	29	27	41	6	
Number of pain sites	No or 1 pain site	4 (11.43%)	2 (6.90%)	3 (11.11%)	11 (26.83%)	0 (0.00%)	*p* = 0.29
	2 pain sites	10 (28.57%)	11 (37.93%)	9 (33.33%)	12 (29.27%)	2 (33.33%)	
	3 pain sites	14 (40.00%)	9 (31.03%)	12 (44.44%)	12 (29.27%)	1 (16.67%)	
	4 pain sites	7 (20.00%)	7 (24.14%)	3 (11.11%)	6 (14.63%)	3 (50.00%)	

*p*—Qualitative variables: chi-squared or Fisher’s exact test. Quantitative variables: Kruskal–Wallis test + post hoc analysis (Dunn test). * Statistically significant (*p* < 0.05).

**Table 7:** Pain characteristics according to the #ENZIAN B classification.

Parameter		B0 (N = 6)	B1 (N = 17)	B2 (N = 38)	B3 (N = 77)	*p*
Duration of pain associated with endometriosis	No pain	1 (16.67%)	0 (0.00%)	4 (10.53%)	0 (0.00%)	*p* = 0.194
	1–4 days in a month	0 (0.00%)	2 (11.76%)	6 (15.79%)	16 (20.78%)	
	5–9 days in a month	1 (16.67%)	5 (29.41%)	5 (13.16%)	17 (22.08%)	
	10–14 days in a month	1 (16.67%)	4 (23.53%)	8 (21.05%)	19 (24.68%)	
	15 or more days in a month	3 (50.00%)	6 (35.29%)	15 (39.47%)	25 (32.47%)	
Intensity of pain in the most severe symptoms of pain in the lower pelvic region [NRS]	Mean (SD)	8.5 (2.74)	8.41 (1.8)	7.53 (2.9)	8.27 (1.74)	*p* = 0.605
	Median (quartiles)	9.5 (9–10)	9 (7–10)	8 (6–10)	9 (8–10)	
	Range	3–10	5–10	0–10	2–10	
	n	6	17	38	77	
Intensity of pain in the most severe symptoms of pain during bowel movements [NRS]	Mean (SD)	6 (3.58)	3.41 (3.26)	4.11 (3.91)	4.22 (3.98)	*p* = 0.625
	Median (quartiles)	6 (5–8.5)	3 (0–5)	4.5 (0–8)	4 (0–8)	
	Range	0–10	0–10	0–10	0–10	
	n	6	17	38	77	
Intensity of pain in the most severe symptoms of pain during urination [NRS]	Mean (SD)	2.5 (3.56)	3.82 (3.68)	1.63 (2.87)	1.31 (2.48)	*p* = 0.027 * B1 > B2, B3
	Median (quartiles)	1 (0–3.5)	5 (0–6)	0 (0–2)	0 (0–1)	
	Range	0–9	0–10	0–9	0–9	
	n	6	17	38	77	
Intensity of pain in the most severe symptoms of pain during or after sexual intercourse [NRS]	Mean (SD)	6.5 (3.21)	6.62 (2.03)	4.68 (3.39)	4.53 (3.17)	*p* = 0.077
	Median (quartiles)	6.5 (4.5–9.25)	6.5 (5.75–8.25)	6 (0.25–7)	5 (2–7)	
	Range	2–10	3–10	0–10	0–10	
	n	6	16	38	76	
Number of pain sites	No or 1 pain site	1 (16.67%)	0 (0.00%)	10 (26.32%)	9 (11.69%)	*p* = 0.012 *
	2 pain sites	0 (0.00%)	4 (23.53%)	7 (18.42%)	33 (42.86%)	
	3 pain sites	3 (50.00%)	6 (35.29%)	15 (39.47%)	24 (31.17%)	
	4 pain sites	2 (33.33%)	7 (41.18%)	6 (15.79%)	11 (14.29%)	

*p*—Qualitative variables: chi-squared or Fisher’s exact test. Quantitative variables: Kruskal–Wallis test + post-hoc analysis (Dunn test). * Statistically significant (*p* < 0.05).

**Table 8:** Pain characteristics according to the #ENZIAN C classification.

Parameter		C0 (N = 58)	C1 (N = 13)	C2 (N = 28)	C3 (N = 39)	*p*
Duration of pain associated with endometriosis	No pain	2 (3.45%)	0 (0.00%)	0 (0.00%)	3 (7.69%)	*p* = 0.116
	1–4 days in a month	6 (10.34%)	1 (7.69%)	6 (21.43%)	11 (28.21%)	
	5–9 days in a month	10 (17.24%)	3 (23.08%)	10 (35.71%)	5 (12.82%)	
	10–14 days in a month	14 (24.14%)	4 (30.77%)	7 (25.00%)	7 (17.95%)	
	15 or more days in a month	26 (44.83%)	5 (38.46%)	5 (17.86%)	13 (33.33%)	
Intensity of pain in the most severe symptoms of pain in the lower pelvic region [NRS]	Mean (SD)	8.53 (1.61)	7.92 (2.02)	7.93 (2.11)	7.62 (2.86)	*p* = 0.433
	Median (quartiles)	9 (8–10)	8 (8–9)	8 (7–9)	8 (6–10)	
	Range	4–10	4–10	0–10	0–10	
	n	58	13	28	39	
Intensity of pain in the most severe symptoms of pain during bowel movements [NRS]	Mean (SD)	3.91 (4.04)	3.77 (3.72)	3.43 (3.33)	5.21 (3.89)	*p* = 0.23
	Median (quartiles)	3 (0–8)	4 (0–6)	3.5 (0–5.25)	6 (0–8.5)	
	Range	0–10	0–10	0–10	0–10	
	n	58	13	28	39	
Intensity of pain in the most severe symptoms of pain during urination [NRS]	Mean (SD)	2.55 (3.34)	1.54 (2.26)	1.11 (2.28)	1.13 (2.53)	*p* = 0.046 * C0 > C3, C2
	Median (quartiles)	0 (0–5)	0 (0–4)	0 (0–0)	0 (0–0)	
	Range	0–10	0–5	0–7	0–8	
	n	58	13	28	39	
Intensity of pain in the most severe symptoms of pain during or after sexual intercourse [NRS]	Mean (SD)	5.28 (3.13)	4.38 (3.91)	4.54 (2.67)	4.79 (3.4)	*p* = 0.661
	Median (quartiles)	6 (3–8)	5 (0–7)	5 (2.75–6.25)	5 (2–8)	
	Range	0–10	0–10	0–9	0–10	
	n	57	13	28	38	
Number of pain sites	No or 1 pain site	8 (13.79%)	2 (15.38%)	4 (14.29%)	6 (15.38%)	*p* = 0.491
	2 pain sites	13 (22.41%)	6 (46.15%)	12 (42.86%)	13 (33.33%)	
	3 pain sites	22 (37,93%)	5 (38.46%)	8 (28.57%)	13 (33.33%)	
	4 pain sites	15 (25.86%)	0 (0.00%)	4 (14.29%)	7 (17.95%)	

*p*—Qualitative variables: chi-squared or Fisher’s exact test. Quantitative variables: Kruskal–Wallis test + post-hoc analysis (Dunn test). * Statistically significant (*p* < 0.05).

**Table 9:** Pain characteristics according to bladder involvement (FB) in the #ENZIAN classification.

Parameter		FB Bladder		*p*
		No (N = 131)	Yes (N = 7)	
Duration of pain associated with endometriosis	No pain	4 (3.05%)	1 (14.29%)	*p* = 0.243
	1–4 days in a month	23 (17.56%)	1 (14.29%)	
	5–9 days in a month	27 (20.61%)	1 (14.29%)	
	10–14 days in a month	29 (22.14%)	3 (42.86%)	
	15 or more days in a month	48 (36.64%)	1 (14.29%)	
Intensity of pain in the most severe symptoms of pain in the lower pelvic region [NRS]	Mean (SD)	8.21 (2.09)	5.86 (2.79)	*p* = 0.021 *
	Median (quartiles)	9 (7.5–10)	5 (4.5–7.5)	
	Range	0–10	2–10	
	n	131	7	
Intensity of pain in the most severe symptoms of pain during bowel movements [NRS]	Mean (SD)	4.34 (3.86)	1 (1.91)	*p* = 0.029 *
	Median (quartiles)	5 (0–8)	0 (0–1)	
	Range	0–10	0–5	
	n	131	7	
Intensity of pain in the most severe symptoms of pain during urination [NRS]	Mean (SD)	1.72 (2.88)	2.57 (3.15)	*p* = 0.251
	Median (quartiles)	0 (0–3)	1 (0–4.5)	
	Range	0–10	0–8	
	n	131	7	
Intensity of pain in the most severe symptoms of pain during or after sexual intercourse [NRS]	Mean (SD)	4.98 (3.17)	3.43 (3.26)	*p* = 0.228
	Median (quartiles)	5 (2–7)	5 (0–6)	
	Range	0–10	0–7	
	n	129	7	
Number of pain sites	No or 1 pain site	18 (13.74%)	2 (28.57%)	*p* = 0.105
	2 pain sites	41 (31.30%)	3 (42.86%)	
	3 pain sites	48 (36.64%)	0 (0.00%)	
	4 pain sites	24 (18.32%)	2 (28.57%)	

*p*—Qualitative variables: chi-squared or Fisher’s exact test. Quantitative variables: Mann–Whitney test. * Statistically significant (*p* < 0.05).

**Table 10:** Pain characteristics according to intestinal involvement (FI) in the #ENZIAN classification.

Parameter		FI Intestinum		*p*
		No (N = 103)	Yes (N = 35)	
Duration of pain associated with endometriosis	No pain	3 (2.91%)	2 (5.71%)	*p* = 0.927
	1–4 days in a month	18 (17.48%)	6 (17.14%)	
	5–9 days in a month	21 (20.39%)	7 (20.00%)	
	10–14 days in a month	25 (24.27%)	7 (20.00%)	
	15 or more days in a month	36 (34.95%)	13 (37.14%)	
Intensity of pain in the most severe symptoms of pain in the lower pelvic region [NRS]	Mean (SD)	8.14 (2.2)	7.97 (2.13)	*p* = 0.555
	Median (quartiles)	9 (7–10)	8 (7.5–10)	
	Range	0–10	2–10	
	n	103	35	
Intensity of pain in the most severe symptoms of pain during bowel movements [NRS]	Mean (SD)	3.74 (3.73)	5.43 (3.98)	*p* = 0.025 *
	Median (quartiles)	4 (0–8)	6 (0–9)	
	Range	0–10	0–10	
	n	103	35	
Intensity of pain in the most severe symptoms of pain during urination [NRS]	Mean (SD)	2.01 (3.08)	1.03 (2.12)	*p* = 0.18
	Median (quartiles)	0 (0–5)	0 (0–0.5)	
	Range	0–10	0–7	
	n	103	35	
Intensity of pain in the most severe symptoms of pain during or after sexual intercourse [NRS]	Mean (SD)	4.95 (3.24)	4.77 (3.06)	*p* = 0.707
	Median (quartiles)	6 (3–7)	5 (2–7)	
	Range	0–10	0–10	
	n	101	35	
Number of pain sites	No or 1 pain site	16 (15.53%)	4 (11.43%)	*p* = 0.451
	2 pain sites	32 (31.07%)	12 (34.29%)	
	3 pain sites	33 (32.04%)	15 (42.86%)	
	4 pain sites	22 (21.36%)	4 (11.43%)	

*p*—Qualitative variables: chi-squared or Fisher’s exact test. Quantitative variables: Mann–Whitney test. * Statistically significant (*p* < 0.05).

## Data Availability

No new data were created or analyzed in this study. Data sharing is not applicable to this article.

## Abbreviations

The following abbreviations are used in this manuscript:

ASRM	American Society for Reproductive Medicine
rASRM	Revised American Society for Reproductive Medicine classification
#ENZIAN	ENZIAN classification system for deep infiltrating endometriosis
NRS	Numerical Rating Scale
QoL	Quality of life
BMI	Body mass index
SD	Standard deviation
SEF	Stiftung Endometriose-Forschung
m	Missing or unspecified value
x	Not assessed
